# Primary health care staff's perceptions of childhood tuberculosis: a qualitative study from Tanzania

**DOI:** 10.1186/1472-6963-12-6

**Published:** 2012-01-09

**Authors:** Stephanie Bjerrum, Michala V Rose, Ib C Bygbjerg, Sayoki G Mfinanga, Britt P Tersboel, Pernille Ravn

**Affiliations:** 1 Department of Infectious Diseases, Hvidovre University Hospital, Kettegaards Allé 30, 2650 Hvidovre, Copenhagen, Denmark; 2 Department of International Health, Immunology and Microbiology, University of Copenhagen, Faculty of Health Sciences, Øster Farimagsgade 5,1014 Copenhagen, Denmark; 3 Muhimbili Medical Research Centre, National Institute for Medical Research, P.O. Box 3436, Dar es Salaam, Tanzania; 4 Department for Infectious Diseases, Herlev University Hospital, Herlev Ringvej 75, 2730 Herlev, Denmark

## Abstract

**Background:**

Diagnosing tuberculosis in children remains a great challenge in developing countries. Health staff working in the front line of the health service delivery system has a major responsibility for timely identification and referral of suspected cases of childhood tuberculosis. This study explored primary health care staff's perception, challenges and needs pertaining to the identification of children with tuberculosis in Muheza district in Tanzania.

**Methods:**

We conducted a qualitative study that included 13 semi-structured interviews and 3 focus group discussions with a total of 29 health staff purposively sampled from primary health care facilities. Analysis was performed in accordance with the principles of a phenomenological analysis.

**Results:**

Primary health care staff perceived childhood tuberculosis to be uncommon in the society and tuberculosis was rarely considered as a likely differential diagnosis. Long duration and severe signs of disease together with known exposure to tuberculosis were decisive for the staff to suspect tuberculosis in children and refer them to hospital. None of the staff felt equipped to identify cases of childhood tuberculosis and they experienced lack of knowledge, applicable tools and guidelines as the main challenges. They expressed the need for more training, supervision and referral feedback to improving case identification.

**Conclusions:**

Inadequate awareness of the burden of childhood tuberculosis, limited knowledge of the wide spectrum of clinical presentation and lack of clinical decision support strategies is detrimental to the health staff's central responsibility of suspecting and referring children with tuberculosis especially in the early disease stages. Activities to improve case identification should focus on skills required by primary health care staff to fulfil their responsibility and reflect primary health care level capacities and challenges.

## Background

Tuberculosis (TB) in children is a serious condition, and in endemic regions TB is a likely cause of death among children with symptoms of respiratory infection [1]. The relative proportion of TB cases occurring in children is found to vary significantly between countries [2], and Marais et al. have estimated that children are likely to represent 15-20% of the disease burden in areas where the TB epidemic is poorly controlled [1]. Diagnosing TB in children is complex and challenged by investigations methods being inaccessible in resource-poor areas [1,3-5]. TB diagnosis is often delayed and repeated visits to the same level of health care, especially at primary facility level, without correct assessment of the patient has been identified as a core reason for this delay among adults [6,7].

In Tanzania, as well as many other TB endemic countries, Primary Health Care (PHC) staff are responsible for identifying children with symptoms suggestive of TB, and referring them to secondary health care level for confirmation of diagnosis and treatment [8,9]. Despite this fact, few studies have explored the associated challenges and resulting delays from the perspective of PHC staff. This underlines the importance of engaging with health staff at PHC level in order to get a better understanding of how childhood TB is managed and to identify challenges that impede case detection. We chose to use qualitative techniques and focus our study on PHC staff from health centres and dispensary in Muheza District, Tanzania. Our objectives were to explore PHC staff's understanding and perceptions of childhood TB as well as their perceived challenges and needs in identifying children with TB, in order to eventually contribute to improved childhood TB case detection.

### The Tanzanian context

In Tanzania, the estimated total annual incidence of TB was 183/100,000 in 2009 [10]. The notification rate for children was 12/100,000 but incidence estimates for childhood TB are not available. National recommendations for interventions to control TB are found in the Manual of the National Tuberculosis and Leprosy Programme (NTLP), 2006 edition [8]. The manual includes a section on childhood TB diagnosis, treatment and prevention, which is in line with the WHO 2006 guidance for the management of childhood TB [9]. Moreover, the NTLP manual contains a childhood TB score chart that is meant as an aid to systematically guide the clinician to collect relevant diagnostic information.

Dispensaries are the first level in the PHC sector usually staffed by a clinical officer and a nurse. At the level above dispensaries, health centres are normally staffed by an assistant medical officer, several clinical officers, nurses and/or midwives. The clinical officers and assistant medical officers are non-graduate clinicians with respectively 3 and 5 years of clinical training in paramedical collages. TB diagnostic services are available in hospitals located in urban centres. Health care is free for all children under 5 years and TB diagnosis and treatment is free for all.

## Methods

### Study setting

This study was carried out in Muheza district in north-eastern Tanzania, a rural district with a population of 209,480 where children under 5 years account for 21% of the total population. The district has 1 hospital, 3 health centres and 38 dispensaries. The government is the principal health care provider with 1 health centre and 28 dispensaries. Non-Governmental Organizations (NGOs) and private providers manage the remaining facilities [11]. The estimated under-5-mortality rate in 2004 was 208/1,000 [12]. The TB notification rate in the district was 348/100,000 with 47% of TB patients co-infected with HIV [13]. Children under 15 years accounted for 75/761 (9.8%) of new TB cases with 34/75 cases (45%) being ≤ 5 years of age, and 41/75 cases (55%) between 6 and 14 years old. Only 4 cases had a positive smear microscopy [14].

### Approach

We considered a combination of semi-structured interviews (SSIs) and Focus Group Discussions (FGDs) to be the most appropriate method to elicit information about PHC staff's perceptions of childhood TB [15,16]. SSIs were conducted to obtain in-depth information about their understanding of and past experiences with childhood TB. We used the dynamics of FGDs to facilitate critical exploration of the PHC staff's views on challenges and needs related to identifying and referring suspected cases of childhood TB. Moreover, FGDs provided information about staff's interactions and reactions to each other's opinions. The study comprised 13 SSIs and 3 FGDs. Data were collected in April 2010.

### Study population, sampling and participation

Purposive sampling was applied to select health staff from the PHC level. A total of 29 health staff were selected from 28 different PHC facilities, including 25 dispensaries and 3 health centres out of 38 and 3 respectively in the district. Informants were sampled to target front line staff who would be expected to identify and manage suspected cases of childhood TB as part of their daily work. Informants included clinical officers (25), assistant medical officer (1), assistant clinical officer (1) and nurses (2). To reflect the various health care providers in the district informants were recruited from governmental (21), NGO (1) and private sector (6) health facilities. All PHC facilities were located at varying distances from the nearest referral hospital and TB clinic as well as the district administration, which allowed for potentially different perceptions depending on geographical location and provider characteristics. Our sampling approach was used for both interviews and FGDs. We used data saturation criteria to establish the required number of interviews and FGDs, i.e. when additional interviews no longer added any new insight to the collected data, interviewing was stopped on the particular issue under investigation [16].

Participation in the study was voluntary and all informants were requested to give informed consent in writing. De-identification and confidentiality was assured by assigning codes to the interviews and groups discussions. The Tanzanian Medical Research Coordinating Committee (NIMR/HQ/R.8a/Vol IX/584) provided research and ethical clearance of this study.

### Data collection

To guide the SSIs and FGDs we used thematic interview guides with broad and open-ended questions. The guide covered the following main themes; (a) Awareness of childhood TB; (b) Identification and referral of suspected cases of childhood TB; (c) Tools and supportive structures; (d) Challenges and needs. Additional themes that emerged in the early stages of data collection were added to the guide for subsequent interviews. Each SSI was concluded with a short structured and close-ended section to elicit information about available tools and guidelines for management of childhood TB. Participatory Rural Appraisal principles and techniques, such as listing and ranking, were applied during the interviews and FGDs to encourage contribution from all informants and provide impressions of the participants' priorities [15]. If an informant had difficulty in providing options for ranking during the ranking exercise, typical challenges as described in the literature were written on pre-manufactured cards and proposed for the participant to consider. Four to six PHC staff participated in each of the FGDs. Participants in the FGDs included both men and women with different years of experience which may have hampered free exchange of views and experiences. Yet, we experienced lively discussions among participants who appeared to be unaffected by any status difference between them.

A Tanzanian research assistant, fluent in Swahili and English, interpreted all interviews and FGDs, which were conducted and moderated by the first and second authors (SB and MVR). Interviews and FGDs were digitally recorded and an independent research assistant transcribed all recordings and provided a second translation of the interviews in writing. We compared the translations during data collection, and found no significant discrepancies in content. Each day after data collection, we considered potential misunderstandings due to the use of an interpreter and discussed how to ask the most relevant questions in subsequent interviews to encourage free and open responses. This also contributed to clarify the content and identify main themes and issues to be explored in later interviews.

### Data analysis

The data analysis followed the principles of phenomenological analysis [15-18] and the stages of: (1) Reading all the transcripts to get an overall impression; (2) Identifying units of meaning and coding these units; (3) Condensing and summarizing the content of each coded group; and (4) Integrating the insight from the condensed meaning units into a generalized description that reflect apparent significant factors. The frame used for coding was derived from the core themes covered by the interview guide and themes emerging from the initial steps of data analysis. All data was subsequently indexed according to the coding frame and grouped for comparison and contrasting across SSIs and FGDs. During analysis, special attention was given to search for deviant cases that contradicted the emerging themes and their general description [19]. Statements were contrasted and compared with existing research-based knowledge, although to our knowledge the literature on this topic seen from the perspective of PHC staff is scarce.

### Assessing the quality of approaches, methods and data

To assess data quality, we considered respondent validation as well as transparency and careful review of the conditions under which information was gained to be the most important aspects. Respondent validation [20] was applied to the extent of testing and exploring the researchers' interpretation of information gained during initial SSIs and FGDs in subsequent interviews and discussions. Particularly interesting findings from the interviews were introduced during FGDs for validation. The status difference between the researchers and the PHC staff could potentially be experienced as intimidating by the PHC staff. However, the research team sought to establish an accommodating atmosphere by carefully introducing the study, being attentive and avoiding a judgemental approach towards the interviewees, and instead giving them space and positive recognition to tell their story. To reduce the researcher's individual preconceptions and maintain reflexivity [21], the study design, data collection, analysis and report writing was undertaken in collaboration between authors.

## Results

A total of 13 semi-structured interviews and 3 focus group discussions were held with 29 PHC staff from 25 dispensaries and 3 health centres. No major discrepancy was found in the perceptions of staff from the governmental, NGO and private sector. Four main themes were identified, and are outlined below.

### Recognition of childhood TB: Burden of disease and perceptions of the diseases entity

Interviews and group discussions revealed that the perceived prevalence of childhood TB combined with the severity of signs and symptoms in a child presenting at the dispensary or health centre had a decisive influence on whether PHC staff would suspect TB. None of the staff perceived childhood TB as a significant problem in the community, although adult TB was regarded to be common. Respondents agreed that the majority of children presenting at the dispensaries or health centres suffered from illnesses that resolved spontaneously or were cured by short course treatments. The following quote illustrates this:

"No, there are no TB cases for children around here. Actually in this street we only receive adult people, they are many.... But when children come with complaints of chest pains and we give them penicillin and PPF (antimalaria) for 5 days, they survive." (Interview 4)

When respondents were asked about signs and symptoms which would make them suspect childhood TB, they listed the core signs and symptoms i.e. failure to thrive, weight loss, persistent cough and fever. Night sweats, lymphadenopathy and pleural effusion were mentioned occasionally. Respondents often included family history of TB, but rarely included parent's HIV status in their assessment, although it was acknowledged that co-infection with HIV and TB was frequent. The following quote summarizes the general view on signs and symptoms of childhood TB:

"In a child I think the signs may be cough, and another thing is failure to thrive. Also, if I find there is a history of TB in the family maybe the mother or anyone in the family, or in the nearest ten households. Or if they visited somewhere who is sick with TB, or is in TB treatment. So I suspect TB in a child." (Interview 10)

The staff emphasized that they primarily suspected TB among children with severe signs and symptoms, long duration of illness, known contact to an adult TB patient and no response to treatment trials of antimalarial drugs and ordinary antibiotics. It was claimed unproblematic to differentiate TB from diseases with similar clinical presentations like respiratory tract infection, malaria and gastrointestinal infections. TB was typically differentiated from such more common acute diseases, on the basis of the child being unresponsive to treatment and severely sick. Respondents found it hard to distinguish HIV and malnutrition from TB and reported that only the hospital had the necessary expertise to differentiate these conditions from each other. However, HIV and malnutrition was considered to be uncommon in their uptake area.

TB was only rarely suspected in children. Respondents stated that childhood TB was not present in their community, but mentioned that they probably saw very little childhood TB because they were not able to diagnose it. Respondents also stated that caregivers would self-refer severely sick children directly to the hospital, bypassing the PHC level. The following quotes illustrate these perceptions:

"You might miss them because you don't have facilities to testing them." (Interview 10)

"The problem here is, when the parent sees that their children's condition is terrible they are going directly to [Teule hospital]. So we just receive minor cases." (Interview 2)

### Case identification: the process and available tools

The PHC staff reported that, due to the required advanced medical skills and tests, it was the responsibility of the hospital to confirm TB diagnosis and initiate treatment in children. The dispensary and health centre staff felt their responsibility was to identify cases suspect of childhood TB and refer them to the nearest hospital. The following quotes express this:

"We can suspect, but we like to prove the diagnosis by referring to district hospital" (Interview 1)

"Most of them are diagnosed at Teule [Hospital] and then they refer them here for continuation of treatment" (Interview 6)

The staff consistently reported that identification of child TB suspects at dispensary level relied on basic clinical examination, history taking from the patient/caregiver, and treatment trials with antimalarials and antibiotics. The respondents mentioned few tools that could assist them in identifying children with suspect TB. The most frequently mentioned tool was the Mother-Child Health (MCH) card that tracks children's weight over time. Some had access to a microscope, which could be used to exclude malaria. HIV rapid tests were available at ten out of thirteen dispensaries, but our respondents made no use of these tests in the assessment of TB suspects. From the close-ended questionnaire it appeared that none of the respondents had been introduced to childhood TB score charts or made notice of the score chart in the NTLP Manual. Half of the respondents (5/10) from the governmental sector and none from the NGO or private sector had received the NTLP Manual. No one was aware of the 2006 WHO guidelines for management of tuberculosis in children. TB registers and TB treatment cards were available at all governmental health facilities, but not at any of the NGO or privately managed facilities. The following quote summarizes the process of identification of children suspected for having TB:

"I can diagnose a child with TB by taking history from the biological mother. I will also do physical observation; I will take the MCH card to check for the weight, and I will do general examination. Afterwards, I will ask the mother some questions." (FGD 1)

The respondents mentioned that it was difficult to obtain a good medical history because of children's inability to articulate symptoms. Medical history was obtained from caregivers, and staff were of the opinion that the information could be biased by ignorance and stigma surrounding the TB diagnosis and possible HIV co-infection. The respondents explained that medical history taking was further challenged by lack of an appropriate recording system to keep track of patient histories, visits to the health facility and prior treatments. Moreover, respondents expressed frustrations about inadequate staffing that prevented thorough history taking and clinical examination of children.

During one FGD, respondents spontaneously discussed the need for active case finding strategies to identify more children with TB as indicated by the following quote:

"Maybe we have to make sure to screen those families with a TB patient to see the possibility of other infected.... We can do it, but not efficiently due to our geography. You see, some patient lives very far away and I don't have transport to visit them. Maybe if we could tell them to come with their families for screening in our health centres, but to go to them it is so difficult." (FGD 2)

However, respondents were generally not familiar with the concept of contact tracing or the programmatic expectations in relation to this. As illustrated in the quote above, the respondents foresaw geographical distance between the PHC facility and the patients to be problematic for their role in active case finding. Other obstacles mentioned in relation to contact tracing were limited tools and staff capacity.

### Referral

Only 2 staff stated that they often referred children suspect of TB, whilst the rest of the staff rarely made referrals to the nearest hospital for investigation. We probed for problems related to referral, and according to respondents' knowledge, the most important reason for delayed referral or no referral at all was time caregivers spent away from daily work and transportation fees incurred by the caregivers in case of referral. Others claimed not to observe any referral problems. The following quote illustrates the perceived referral challenges, which, despite being mentioned frequently in our interviews and FGDs, was given low importance in the ranking of all challenges to suspect TB in children.

"Sometimes there are problems because the patient may not be satisfied with that referral and he might take it as disturbance, or he can see it expensive. Sometimes for example you can refer someone, but they stay at home until the patient's condition becomes critical. That is when they decide to go." (FGD 2)

The respondents desired feedback about the patients sent to the hospital for tests and diagnosis, as well as information about any treatment initiated. However, frustration was expressed regarding non-existent or inconsistent referral feedback, and it was not clear to the respondents whose responsibility it was to ensure feedback subsequent to referral. One opinion expressed was that the district hospital/TB unit should give feedback to the dispensaries. Others found that patients should report to the dispensary, or that the staff themselves should follow-up on the patients' status.

"Only some patients who have been discovered to have TB will come back with their cards to continue taking drugs here. But for the majority, we don't get feedback from the district, this is also a challenge." (Interview 8)

### Lack of training, supervision and tools

Respondents did not feel adequately equipped for identifying children suspect of TB. Lack of training and diagnostic tools was ranked as the most significant barrier to identifying children with suspect TB, and training of staff was perceived to be the most important intervention to improve performance at PHC level. In-depth discussions revealed that few had received training in TB since college, and no one had specific training in childhood TB. Moreover, support and supervision was perceived to be inadequate and a missed opportunity for education, continuous training and for motivating the health staff at dispensaries and health centres. The following quotes illustrate the expectations for change in these areas:

"TB patients they don't come here every day. So if you don't practice anti-TB, sign and symptoms of TB, then you forget. So there is a need to be reminded every now and then. I mean to refresh your mind about TB treatment, signs and symptoms and something like that." (Interview 6)

"DTLC [District Tuberculosis and Leprosy Coordinator] he visits two to three times a year, few very few. It doesn't help us much, because when he comes he specifically comes to reconcile with your TB patients' record book. And he will take his recording book and write his own report and that is it. To visit maybe during the RCH [Reproductive Child Health Clinics], there will be a possibility that we can screen the children together and this will help me getting skills." (FGD2)

Respondents emphasized the need for simple diagnostic tools and guidelines for screening children and improving TB case detection. Positive interest was shown towards childhood TB score charts and a flowchart similar to the format of the Integrated Management of Childhood Illnesses (IMCI) guidelines and posters. The following quote illustrates the demand for better diagnostic tools:

"If we could have this special investigation for the unit that we can apply to that kid directly. A simple one not complicated as this x-ray, maybe it could help us" (FGD 3)

## Discussion

### What does this study add to previous knowledge?

Qualitative analysis of 13 SSI and 3 FGDs with PHC staff suggest that childhood TB is rarely suspected and not perceived to constitute an important health problem in the study area. While this finding could reflect a genuine low burden of childhood TB in the population, it conflicts with previous data, which indicates a rising burden of total TB with evidence of children contributing significantly to the total TB caseload in high-endemic communities [1,2,22]. We find it more likely that our finding reflects an inadequate awareness of childhood TB in the study setting. Most of the staff in our study had not received specific education or guidelines with regards to childhood TB, although such guidance is essential to create awareness and ensure implementation of expected practices. Nor did the staff receive feedback from the hospital subsequent to referral of suspected childhood TB cases, leaving them unaware of the burden of TB disease suffered by children. Retrospective studies support this finding, showing that reports of children diagnosed with TB at referral hospitals do not always get back to the primary care clinics [22,23].

Childhood TB cannot be viewed as a single disease entity and the natural history of TB, as it is described in the pre-chemotherapy literature, comprises a wide spectrum of manifestations [24]. Our study revealed that PHC staff had a uniform expectation that children with TB present with a long history of disease, classic and obvious symptoms of intra-thoracic TB combined with apparent risk factors. TB was rarely considered as a differential diagnosis, despite the fact that TB can mimic many common childhood diseases including pneumonia, generalized bacterial and viral infections, malnutrition and HIV. We argue that this indicates a missed opportunity for childhood TB case detection, as childhood TB is not suspected in the early stages of the disease where symptoms are less severe and more general. Moreover, our findings show that TB will rarely be considered in children with unknown exposure to TB, or in more complicated situations with haematogenous spread and extra-pulmonary TB.

The PHC staff identified the most important needs for improving identification of children with TB at PHC level to be training, better tools and appropriate guidelines. Our findings show that the health staff were well informed about the core symptoms and risk factors for childhood TB, but not the extent of the childhood TB burden and wide spectrum of clinical presentations. We support efforts to train health staff, but strongly argue that the focus of training should be in accordance with the responsibilities of PHC level health staff, i.e. to suspect and refer cases. Based on our findings, training should aim at increasing awareness of the burden of childhood TB backed up by the best available epidemiological information, and expand the knowledge of clinical signs and symptoms of the disease with emphasis on early symptoms and timely referral of these children.

Our study also identified a missed opportunity for continuous learning and motivation of PHC staff through strengthening of supervision and feedback systems. The staff did not feel that the present supervision of their work was adequate or supportive. They were further demotivated by not receiving feedback about their work or the outcome of patients referred to hospital. Another study from Tanzania describes absence of referral feedback to PHC level as a general constraint to quality health service delivery and motivation of PHC health staff [25]. TB registers and treatment cards were not available at the privately or NGO managed health facilities included in this study, which indicates a serious breach in the chain of providing referral feedback to these health care providers.

Training, positive and more systematic supervision as well as structured feedback systems have previously been emphasized as important areas to develop health workers' clinical skills, increase job satisfaction and reduce diagnostic delay [7,25,26]. However, a number of studies that evaluate IMCI have demonstrated difficulties of implementing effective training and supervision, even though IMCI is relatively well institutionalized in national health systems [27-29].

Better diagnostic modalities have been the target of much research [1,3,4]. In response to the diagnostic challenges, a variety of clinical scoring systems have been developed to assess child TB cases. These clinical scoring systems are meant to provide health staff in resource poor settings with a rational, stepwise and feasible aid for childhood TB screening, but are often criticized for lacking standard symptom definitions and adequate validation [30-32]. While the PHC staff in our study requested simple tools and guidelines to identify child TB suspects, the clinical score chart included in the Tanzanian NTLP Manual, was unknown to the staff in our study. Moreover, the score chart is partly based on x-ray and other investigation tools, which are unavailable to the PHC staff and hence not adapted to their setting and responsibilities. An assessment report of the management of childhood TB in Tanzania confirms these findings [33]. It is evident that clinical decision support systems must be targeted the level of use and only be based on applicable tools. We argue that tools and guidelines made available at PHC level must reflect the responsibilities and capacities of the PHC staff and take into account the current challenges reported by PHC staff e.g. limited knowledge of childhood TB, limited access to diagnostic tools, inadequate staffing and stigma associated with TB. We recommend an assessment of various strategies for clinical decision support at PHC level e.g. by integrating childhood TB into the existing IMCI guidelines with due attention to evidence from Tanzania and South Africa of limited adherence to IMCI guidelines [34,35].

Low referral rate of paediatric cases from rural facilities to hospital has previously been shown in Tanzania [34,36]. In our study, referral of child TB suspects was a rare event and supposedly compromised by patient incurred costs associated with referral to hospital e.g. transportation fees and time lost from work. A study from Ethiopia confirmed that costs related to TB diagnosis incurred by patients and their families represent a significant proportion of their monthly income [37]. They conclude that health providers' low level of TB suspicion in adult cases results in several consultations at various health facilities before referral and diagnosis, increasing cost of TB diagnosis significantly. This underpins the need for strengthening the PHC staff's skills in early suspicion of childhood TB on the basis of moderate symptoms and appropriate referral of suspected TB cases, while limiting unnecessary referral and overburdening of the National TB programme with unfounded TB suspects.

Contact tracing may be a powerful strategy for improving case detection, and early identification of children with TB [38,39]. This is recognized by WHO which recommends active tracing of young children in close contact with a source case of pulmonary TB as a desired element in the identification of new childhood TB cases [9]. In acknowledgement of the limited resources available for such initiatives, WHO has suggested that a prophylaxis register, keeping track of contacts, should be added within the structures of the country NTLP. The PHC staff in our study setting suggested a similar strategy, but only when confronted with the issue directly, and there were no on-going initiatives with regards to contact tracing. Given the many obstacles previously identified in implementing active case finding and preventive treatment [39,40], we find it important to formulate a specific and detailed strategy of contact tracing within the NTLP.

### Limitations to the study

This study represents the views and experiences of PHC staff from one specific geographical area in Tanzania, which limits the scope for drawing general conclusions. However, the findings are characterized by in-depth and rich information with a range of different perspectives from typical health staff working at Governmental, NGO and private PHC facilities. This makes us confident that our findings can be translated to settings comparable to our study setting. We realise that the researchers' professional status as medical doctors was likely to yield answers that focused on the biomedical aspects of childhood TB rather than the processes of case identification. It is moreover possible that the informants may have avoided exposure of their challenges and sought to produce what they considered as correct answers in order to please the researches. This may explain why it proved difficult to elicit answers beyond standard answers concerning the weaknesses and challenges in case identification, particularly in the SSIs. We found the FGDs more useful in exploring these more sensitive and pertinent issues.

The use of an interpreter can influence the quality of information gained. This was sought reduced by preparing the interpreter before data collection to ensure that he had knowledge about the purpose of the study, his role in the study and the information that interview questions aim to elicit.

To add further quality to the findings, our study could have benefited from direct observation of the health staff at their work site to triangulate between what was said about a situation and what can otherwise be known or experienced concerning this experience. The time commitment required to do this was not possible in this study, but observation of participants is recommended for follow-up studies.

## Conclusions

Primary health care staff perceives childhood TB to be a rare disease and expect children with TB to present with pronounced signs and symptoms together with known exposure to TB. We believe this provides a crucial barrier to childhood TB case detection, and to PHC staff's intended role of suspecting and referring children with TB in the early stages of disease. Based on the PHC staff's expressed needs, TB case detection can be improved if training and supervision activities are strengthened and focused on the critical skills required by them. Referral feedback from hospital to PHC level can further serve to motivate the staff and increase awareness of the childhood TB burden. Tools and guidelines to assist the PHC staff in suspecting and referring cases of childhood TB were neither widely available nor adapted to fit the capacity at PHC level. This calls for clinical decision support strategies, which are specifically designed for the PHC level and answer the challenges reported by PHC staff in our study.

## Competing interests

The authors declare that they have no competing interests.

## Authors' contributions

SB designed the study, collected data, carried out the interviews and FGDs, analysed data and drafted paper. MVR designed and coordinated the study, contributed significantly to data collection, carrying out interviews and FGDs, data analysis and drafting of paper. SB and MVR reached consensus as regards coding. ICB contributed to designing the study, interpretation of data and provided critical feedback on preliminary analysis and draft of paper. SGM contributed to designing the study, interpretation of data and provided critical feedback on draft of paper. BPT contributed to designing the study, interpretation of data and provided critical feedback on draft of paper. PR participated in the conception, design and coordination of the study, contributed to interpretation of data and provided critical feedback on preliminary analysis and draft of paper. All authors read and approved the final manuscript

## Pre-publication history

The pre-publication history for this paper can be accessed here:

http://www.biomedcentral.com/1472-6963/12/6/prepub
